# Cytogenetics Is a Science, Not a Technique! Why Optical Genome Mapping Is So Important to Clinical Genetic Laboratories

**DOI:** 10.3390/cancers15225470

**Published:** 2023-11-19

**Authors:** Adam C. Smith, Alexander Hoischen, Gordana Raca

**Affiliations:** 1 Department of Laboratory Medicine and Pathobiology, Temerty Faculty of Medicine, University of Toronto, Toronto, ON M5S 1A8, Canada; 2 Department of Human Genetics and Department of Internal Medicine, Research Institute for Medical Innovation, Radboud Expertise Center for Immunodeficiency and Autoinflammation and Radboud Center for Infectious Disease (RCI), Radboud University Medical Center, 6525 Nijmegen, The Netherlands; alexander.hoischen@radboudumc.nl; 3 Department of Pathology and Laboratory Medicine, Children’s Hospital Los Angeles, Los Angeles, CA 90027, USA; graca@chla.usc.edu

Karyotyping is a technique that has been used in clinical cytogenetic laboratories for more than 40 years. It is, to this day, a powerful view of the full human genome, still providing new insights into the biology of many human genetic diseases, especially cancer. As a technique, it seems deceptively simple, boiling down to dropping nuclei on a slide, staining the nuclear material, and analyzing metaphases through the microscope. However, as experienced by many laboratories that routinely use karyotyping, this apparently simple process can be very challenging. It requires extensive training and expertise to achieve high-quality preparations, to accurately analyze metaphases, and to report a clinical-grade karyotype. Nevertheless, karyotype analysis has endured for decades as a broadly used clinical assay, as it also provides unique advantages. It is one of the few techniques that maintain an intact genome (without requiring fragmentation and remapping back to a reference) and allows for the detection of both structural and numeric genomic changes. It is also one of the few techniques that directly assesses the ploidy of the nucleus, enabling an actual count of chromosomes in each metaphase.

Conventional cytogenetic techniques are a ‘scientific art’. Cytogenetics requires a commitment to its art to achieve proficiency, which is progressively being lost as cytogeneticists retire and technologies in the laboratory change [1,2]. There is also a prevalent perception that ‘cytogenetics is dying’ and could be replaced by sequencing-based technologies. Many of us who try to recruit technologists into clinical laboratories find the task exceedingly difficult because of this perception.

In addition to requiring hard-to-maintain skills and ‘artistry’, karyotyping has other shortcomings as the primary technique to study chromosomes. Its ability to identify and resolve small and complex rearrangements is very limited [3]. Complex rearrangements ascertained via karyotyping are often reduced to subjective or vague descriptions, such as “add”—a material of unknown origin added to an identifiable chromosome segment, or “mar”—an unidentifiable “marker” chromosome. Karyotyping often requires the ancillary confirmation of observed abnormalities to verify that the breakpoints determined using visual examination indeed involve critical genes. ‘Phenocopies’ or ‘mimics’ are a known phenomenon in cancer cytogenetics; they appear under the microscope as recurrent oncogenic rearrangements but do not involve the relevant genes. Reported examples include mimics of the t(9;22) and t(8;21) that appear to be classic rearrangements but, upon ancillary investigation, were shown not to involve the *BCR* and *ABL1* or *RUNX1* and *RUNX1T1* genes, respectively. While this phenomenon was thought to be rare, the first study of this kind ascertained that ~1% of the rearrangements reported as recurrent oncogenic abnormalities might be non-functional mimics (A. Dubuc, personal communication). This highlights another area where the limits of karyotype analysis can have serious clinical implications.

Cytogeneticists are the first to recognize and acknowledge the limitations of karyotype analysis, seeking and enthusiastically embracing more advanced tools to study chromosomes and expand cytogenetic knowledge. Starting in the 1990s, the implementation of fluorescence in in situ hybridization (FISH) allowed for routine diagnostics and better characterization of classic microdeletion syndromes, the detection and study of subtelomeric rearrangements, and the diagnosis of cancer abnormalities in interphase cells. In the 2000s, chromosomal microarray analysis was embraced as a powerful tool for the high-resolution genome-wide detection of copy number variants (CNVs) and, when combined with genotyping probes (SNP array), also allowed for the genome-wide detection of regions of homozygosity (ROH). Widespread CMA use led to the discovery of numerous microdeletion and microduplication syndromes, recurrent and clinically significant copy number abnormalities in cancer, and a benign copy number variation in human populations [4,5,6]. As a result, the cytogenetic workup for many patients with suspected hematologic malignancies has expanded beyond karyotyping and may include several ancillary techniques, including FISH, CMA, MLPA, and RT-PCR.

## 1. A Cytogenomic Revolution: Looking for Structural Variation at High Resolution

**Figure 1:**
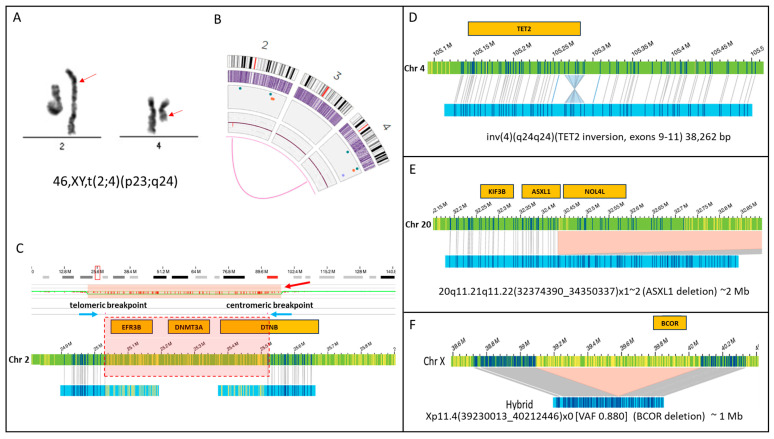
SVs that are undetectable using karyotype analysis. This figure shows four examples of SVs that can disrupt gene function. (**A**) Karyogram showing an apparently balanced translocation involving chromosomes 2 and 4 with breakpoints estimated at bands 2p23 and 4q24 (red arrows) (**B**) Optical Genome Mapping also shows the (2;4) (the magenta line connecting chromosomes 2 and 4 on circos plot). (**C**) However, an evaluation of chromosome 2 breakpoints for this translocation in high resolution shows that they are separated by approximately 500 kilobases, and the intervening sequence is deleted. Blue arrows show the telomeric and centromeric breakpoints. The copy number plot shows a decrease in copy number (red bar), and the region of copy number loss is highlighted by a red box showing the genes that are deleted within this region. Notably, this deletion includes genes of significance in the pathogenesis of hematologic malignancies, like *DNMT3A.* Below the chromosome 2 reference sequence (green bar), the maps for each translocation are shown (blue bars) that map partially to chromosome 2 (matchlines) versus the part of the map that aligns to chromosome 4 (part with yellow label lines). This deletion is not detected by karyotyping. The genes involved are also unknown. (**D**) This SV shows an intra-genic inversion within the coding sequence of *TET2*. This SV likely disrupts *TET2* expression and is too small to be seen via karyotype as it is only 38 kb in size. This SV was also not detected using Myeloid NGS panel testing. (**E**) A 2 Mb deletion involving the 3′ end of the *ASXL2* gene on chromosome 20q. This deletion is too small to be seen via karyotype. (**F**) A one Mb deletion on the X chromosome in a male resulted in the complete deletion of the BCOR gene in 88% of the sample.

Genome mapping and sequencing techniques are emerging as powerful new tools for the research and clinical investigation of genome-wide structural variations (SV). Optical Genome Mapping (OGM) is based on imaging ultra-long (>150 kbp) DNA molecules labeled at a specific 6 bp sequence motif (CTTAAG) that occurs on average every 6 kb across the entire genome. A comparison of the location of the labels between the test sample and a reference genome enables the detection of different classes of SVs, including both copy number changes (deletions, duplications, insertions) and balanced genomic rearrangements (inversions and translocations). In addition, a separate coverage-based algorithm detects large CNVs and aneuploidies. OGM detects all classes of SVs in a single assay and combines the diagnostic capacity of the karyotype, FISH, and CMA. Numerous studies performed on both constitutional and oncology samples have shown the ability of OGM to detect not only SVs identified previously by a combination of other cytogenomic assays but to also reveal additional clinically significant abnormalities [7,8,9,10,11,12]. OGM has already shown that submicroscopic SVs (e.g., deletions, inversions, insertions) that are smaller than 5 Mb and overlap critical genes involved in leukemogenesis are highly under-ascertained with the current testing. A recent study by Collins et al. [13] estimated that up to 25% of deleterious mutations in the human genome may be a result of SVs. Our own data show that many important genes whose involvement significantly alters the diagnostic or prognostic classification of patients are disrupted in tumor samples by SVs, which are undetectable using karyotype analysis (Figure 1).

**Figure 2:**
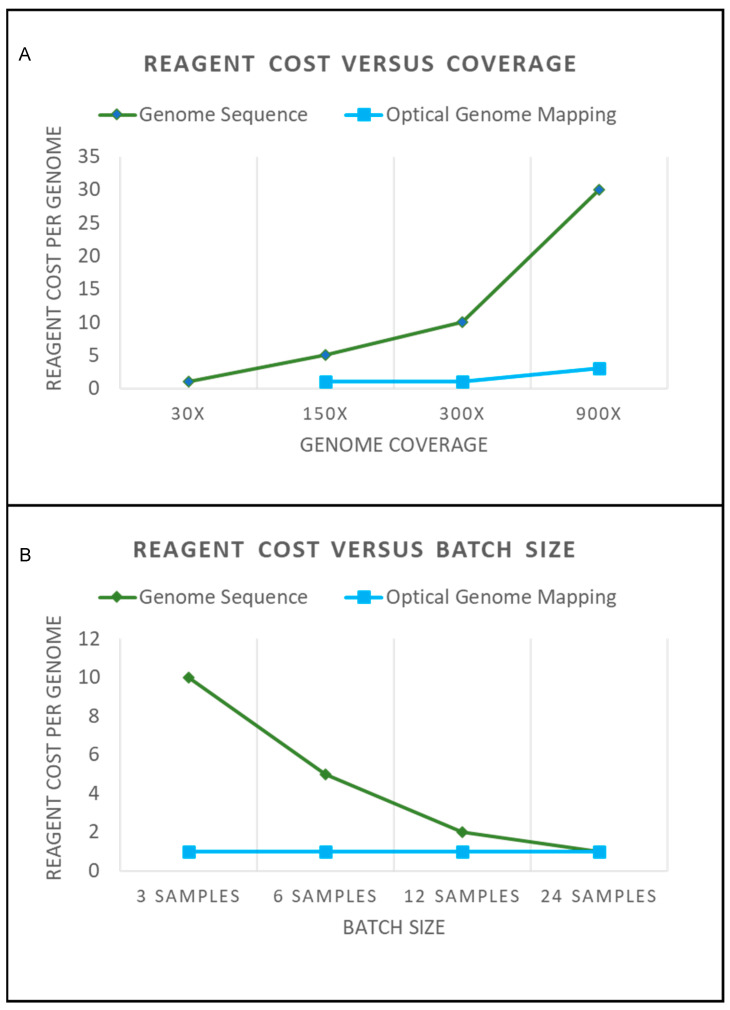
(**A**) The graph shows a general approximation of reagent costs for genome sequencing and optical genome mapping. A hypothetical reagent cost of “1” is shown for a 30× genome sequence that would be required to perform structural variation analysis. A 30× genome sequence would provide adequate coverage for constitutional SVs present in every cell. Mosaic alterations would require additional coverage for detection. The graph shows that the additional coverage for genome sequencing requires additional sequencing. Achieving an adequate coverage depth with a high probability of detecting low-frequency SV would require a >300× coverage. This significantly increases the cost of performing genome sequencing. Conversely, Optical Genome Mapping can be performed from 150× to >400× coverage on a single flow cell. Obtaining a 900× or greater coverage requires letting the sample run longer on the instrument and may require merging data from multiple flow cells (which would increase reagent cost). (**B**) Sequencing costs are also affected by batch sizes. To have an optimal turnaround time and sequencing costs, flow cells need to have an adequate number of samples. As can be seen by the graph, as a hypothetical sequencing flow cell reaches capacity, the cost per sample to sequence becomes cost-effective. However, samples that require urgent turnaround times for reporting cannot always be batched effectively. By contrast, OGM can be run with just three samples, and the cost to run does not change. This makes Optical Genome Mapping ideal for small-to-medium size laboratories, and larger throughput instruments are available to increase scalability (also with no change to the reagent cost). Note that no consideration is made for “volume discounting”, which often happens when assays are run at high volumes. This graph is merely illustrative of how the concepts of coverage and batching affect operational costs.

Short-read genome sequencing (srGS) has demonstrated the ability to detect genome-wide structural variants (SVs) at a cytogenomic scale (larger than 500 bases). However, due to challenges with accurately mapping short reads to the reference genome, the ability of srGS to resolve complex rearrangements and SVs involving large repetitive elements is inferior to OGM [14,15]. Studies that have suggested that srGS can replace conventional cytogenetics as the primary assay for SVs in tumor samples suffer from considerable drawbacks; they only include a targeted evaluation for known recurrent abnormalities, their resolution for the detection of CNVs is limited to ~5 Mb, and their ability to identify variants with a low allele fraction is poor at the depth of coverage of 30–50× typically used for srGS [16]. While it is expected that long-read sequencing technologies could improve SV detection, the current throughput, variant calling/visualization software, price per genome, and achieved coverage all require significant improvements for long-read genome sequencing (lrGS) to be considered a first-tier testing option for oncology applications. The detection of low-level variants (both structural and copy number) is, in fact, one of the key challenges for any GS approach. Having an appropriate sequencing depth for somatic applications is expensive and resource-intensive in terms of reagent cost, computing power, and data storage, as illustrated in Figure 2 [17].

## 2. New Cytogenetic Techniques: Clinical, Logistical and Financial Considerations

The implementation of any new technology in clinical laboratories typically raises logistical and financial considerations. From a financial perspective, SV detection using GS approaches is cost-effective only if performed at a large scale; otherwise, costs rise dramatically. By contrast, scalability is one of the key advantages of OGM (Figure 2). For small-to-medium size laboratories (up to 4000 “karyotype” samples per year), OGM can be run within a week on a single instrument (Stratys) and at the same cost whether data collection is performed for constitutional (~100×) or somatic (~350×) applications. OGM also provides an analysis pipeline and software that is visual and relatively easy to learn. By contrast, logistic barriers to the wide implementation of SV detection from GS are significant; for example, there are no widely available clinically validated pipelines for genome-wide SV detection. At present, an analysis of SVs from sr- or lr-GS data requires considerable bioinformatics infrastructure and experience.

It is pivotal to point out that for many hematologic malignancies, their classification and treatment have been defined by the presence of recurrent cytogenetic abnormalities (e.g., AML, MDS, CML, CMML, MF, CLL, MM, B-ALL, etc). Therefore, any technique that attempts to replace karyotyping must demonstrate an ability to efficiently and accurately recapitulate karyotypic findings. While many of the above diseases may be redefined in the future with an improved understanding of the key leukemogenic determinants, technical innovation (technology agnostic criteria), or simply by changes in treatment options that change the priorities for biomarker detection, at the current time, a genome-wide SV assessment (e.g., a karyotype or its equivalent) is essential for clinical management.

## 3. The Future of Cytogenetics

Cytogenetics is a science that deals with the number, structure, and function of chromosomes within the nucleus and the role of chromosome abnormalities in human disease. While the tools we use to assess the structure and function of chromosomes may change, the study of cytogenetics retains its scope and significance. In fact, as our tools improve, the body of knowledge, diagnostic capabilities, and clinical significance of cytogenetics only increases. Cytogenetics is not dying; one can argue that the emergence of OGM as yet another powerful tool in its repertoire further ensures the growth and relevance of our discipline. In fact, we anticipate that OGM, and possibly other long-read technologies, could enable ‘next generation cytogenetics’ with a new generation of cytogeneticists and years of new cytogenetic insights.

This special edition of *Cancers* on the use of OGM for hematologic malignancies is a timely collection of articles that showcase the strengths and growing applications of OGM and their contributions to the expansion of cytogenetic knowledge.
